# Synergistic Crystallization Modulation and Defects Passivation in Kesterite via Anion‐Coordinate Precursor Engineering for Efficient Solar Cells

**DOI:** 10.1002/advs.202405016

**Published:** 2024-07-19

**Authors:** Lijing Wang, Liangli Chu, Zhengji Zhou, Wenhui Zhou, Dongxing Kou, Yuena Meng, Yafang Qi, Shengjie Yuan, Litao Han, Gang Yang, Zhuhua Zhang, Zhi Zheng, Sixin Wu

**Affiliations:** ^1^ Key Lab for Special Functional Materials Ministry of Education National and Local Joint Engineering Research Center for High‐Efficiency Display and Lighting Technology and School of Materials Henan University Kaifeng 475004 China; ^2^ College of Physics and Electronic Engineering Nanyang Normal University Nanyang 473061 China; ^3^ State Key Laboratory of Mechanics and Control for Aerospace Structures Key Laboratory for Intelligent Nano Materials and Devices of Ministry of Education Institute for Frontier Science Nanjing University of Aeronautics and Astronautics Nanjing 210016 China; ^4^ College of Physics Nanjing University of Aeronautics and Astronautics Nanjing 211106 China; ^5^ Inst Surface Micro & Nano Mat Coll Adv Mat & Energy Key Lab Micronano Energy Storage & Convers Mat He Xuchang University Xuchang Henan 461000 China

**Keywords:** coordination engineering, crystallization modulation, cztsse solar cells, defects passivation, kesterite

## Abstract

It has been validated that enhancing crystallinity and passivating the deep‐level defect are critical for improving the device performance of kesterite Cu_2_ZnSn(S,Se)_4_ (CZTSSe) solar cells. Coordination chemistry interactions within the Cu‐Zn‐Sn‐S precursor solution play a crucial role in the management of structural defects and the crystallization kinetics of CZTSSe thin films. Therefore, regulating the coordination environment of anion and cation in the precursor solution to control the formation process of precursor films is a major challenge at present. Herein, a synergetic crystallization modulation and defect passivation method is developed using P_2_S_5_ as an additive in the CZTS precursor solution to optimize the coordination structure and improve the crystallization process. The alignment of theoretical assessments with experimental observations confirms the ability of the P_2_S_5_ molecule to coordinate with the metal cation sites of CZTS precursor films, especially more liable to the Zn^2+^, effectively passivating the Zn‐related defects, thereby significantly reducing the defect density in CZTSSe absorbers. As a result, the device with a power conversion efficiency of 14.36% has been achieved. This work provides an unprecedented strategy for fabricating high‐quality thin films by anion‐coordinate regulation and a novel route for realizing efficient CZTSSe solar cells.

## Graphical abstract

P_2_S_5_ was introduced into the Cu‐Zn‐Sn‐S precursor solution to optimize grain growth and reduce defect density of Cu_2_ZnSn(S,Se)_4_ absorber. The results demonstrated that P_2_S_5_ molecule can coordinate with the metal cation sites of precursor films, especially more liable to the Zn^2+^, thereby effectively passivating the Zn‐related defects. The power conversion efficiency has conspicuously climbed from 11.28% to 14.36%, making it one of the most high‐efficiency kesterite solar cells.

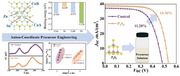

## 1 Introduction

As newly emerging photovoltaic technology, kesterite Cu_2_ZnSn(S,Se)_4_ (CZTSSe) solar cells have developed by leaps and bounds in recent years owing to their earth‐abundant, tunable direct bandgap (1.0–1.5 eV), high optical absorption coefficient (>10^4^ cm^−1^), and environment‐friendly properties.^[^ ^1^, ^2^, ^3^, ^4^, ^5^ ^]^ The optimal power conversion efficiency (PCE) of CZTSSe solar cells have been significantly improved from the initial 0.66%^[^ ^6^ ^]^ to the recently certified 14.9%.^[^ ^7^ ^]^ The rapid progress in PCE is due to tremendous research efforts from the perspective of improving the quality of absorbers,^[^ ^8^, ^9^, ^10^, ^11^, ^12^, ^13^, ^14^, ^15^, ^16^ ^]^ interface engineering,^[^ ^17^, ^18^ ^]^ and optimizing the structure of devices.^[^ ^19^, ^20^ ^]^ Although remarkable improvements have been achieved, the PCE remains significantly below the predicted Shockly‐Quesser (SQ) limit efficiency (≈32%).^[^ ^21^ ^]^ One of the major reasons for this is the large open‐circuit voltage deficit (*V*_OC,def_). It is widely accepted that the larger *V*_OC,def_ is mainly due to the numerous defects in CZTSSe absorbers. Therefore, the development of kesterite thin films with high crystallinity and a low defect density is very important for high‐performance CZTSSe devices.^[^ ^22^ ^]^

Currently, high‐efficiency CZTSSe devices are commonly fabricated by the molecular precursor solution method, the chemical environment and coordination structure in the precursor solution have a direct impact on the crystallization and defect state of CZTSSe absorbers.^[^ ^23^, ^24^, ^25^, ^26^ ^]^ The quality and final optoelectronic properties of the absorbers can be improved by controlling the chemical source composition, the elemental ratio, and different dopants/additives in the precursor solution. For instance, doping of Li^+^, Ag^+^, Cd^2+^, Ge^4+^, and other cations in the precursor solution can reduce defects in CZTSSe absorbers,^[^ ^27^, ^28^, ^29^, ^30^, ^31^, ^32^, ^33^, ^34^ ^]^ regulating the different oxidation states of tin (Sn^2+^ and Sn^4+^) in the precursor solution could optimize the grain growth pathways of the CZTSSe thin films.^[^ ^16^ ^]^ In addition, introducing an appropriate molecular thioglycolic acid (TGA) is able to optimize the precursor coordination structure to promote the subsequent crystal growth process.^[^ ^12^ ^]^ Previous research was mainly focused on improving the composition and chemical environment of the precursor solution by introducing cations. However, anions also have a crucial influence on the chemical coordination of the precursor solution as well as on the composition, energy band structure, and defect state of the final absorbing films. To improve the performance of the CZTSSe photovoltaic devices, it is urgent to further investigate the effect of anions in the precursor solution on the CZTSSe absorbers.

It has been demonstrated that phosphorus (P) incorporation significantly boosts the *V*_OC_ of CdTe devices and enhances the p‐type conductivity of CuInS_2_. In addition, the latest theoretical calculation shows that group VA element P substituting anion in kesterite can entirely passivate the deep‐level defects.^[^ ^35^, ^36^, ^37^ ^]^ These beneficial results show that anion P doping enhances the photovoltaic properties of chalcogenide semiconductors. Herein, phosphorus pentasulfide (P_2_S_5_, P═S group) was successfully used as an anion‐coordinate precursor additive in the Cu‐Zn‐Sn‐S precursor solution. A combination of theoretical calculations and experimental measurements revealed that P_2_S_5_ has strong interaction with metal cation ions of CZTS precursor films, especially for Zn^2+^, which can stabilize the CZTSSe lattice structure and suppress the Zn‐related defects. Finally, the P_2_S_5_ additive solar cells exhibit an outstanding PCE of 14.36%. Our study highlights the synergistic effect of crystallization modulation and defect passivation on the CZTSSe films by optimizing the metal cation coordination structure in the precursor solution for further elevating the performance of CZTSSe solar cells.

## 2 Results and Discussion

**Figure 1:**
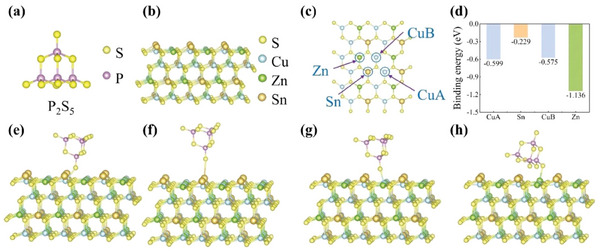
a) Chemical structure of P_2_S_5_. b) The crystal structure of the kesterite CZTS after the incorporation of P_2_S_5_. c) Metal cation binding sites of P_2_S_5_ in CZTS films. d) The binding energies of P_2_S_5_ with metal cation sites. Theoretical simulations of the interaction between P_2_S_5_ and e) CuA, f) Sn, g) CuB, and h) Zn binding sites of CZTS films.

First, density functional theory (DFT) calculations were applied to scrutinize the molecular, electronic, and geometric structures in detail. The ─OH bond is able to react with the P═S bond in P_2_S_5_ to form coordination compounds with metal ions.^[^ ^38^ ^]^ Based on this speculation, we calculate the chemical interaction between P_2_S_5_ and CZTS films. The chemical structure of P_2_S_5_ is shown in **Figure** **1**a. Furthermore, Figures 1b and S1 (Supporting Information) show the crystal structures of CZTS with ideal and actual structures (after the addition of P_2_S_5_), respectively. As illustrated in Figure 1c, the binding sites between P_2_S_5_ and CZTS are CuA, Sn, CuB, and Zn. As shown in Figure 1d, the calculated binding energies of P_2_S_5_ with the above four different sites are −0.599, −0.229, −0.575, and −1.136 eV, respectively. These binding energies are negative, indicating that P_2_S_5_ can exothermically interact with metal ions, especially with Zn^2+^. The corresponding adsorption configurations are shown in Figure 1e–h. Thus, the strong interaction between P═S and metal ions implies that P_2_S_5_ is helpful for a high crystallization CZTSSe thin films, supporting that P_2_S_5_ is promising to reduce the density of Zn‐related defects.

Experimentally, the solution process is considered to be the most commonly used route to prepare high quality CZTSSe absorbers (See the film preparation for more details). P_2_S_5_ additive was added to the (Ag,Cu)_2_ZnSnS_4_ (ACZTS) precursor solution at concentrations of 0, 0.02, 0.05, 0.10, 0.15, and 0.20 mg mL^−1^, respectively (Figure S2, Supporting Information).

**Figure 2:**
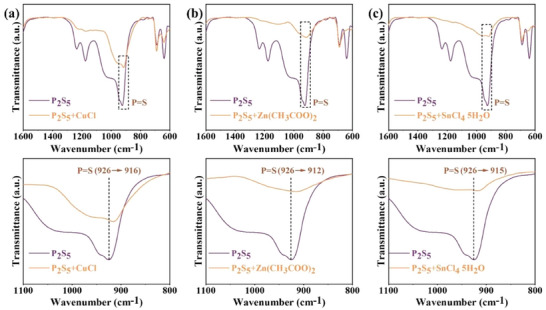
FTIR spectra of a) P_2_S_5_ and P_2_S_5_+CuCl, b) P_2_S_5_ and P_2_S_5_+Zn(CH_3_COO)_2_, and c) P_2_S_5_ and P_2_S_5_+SnCl_4_ 5H_2_O.

To clarify the interaction of P_2_S_5_ additive with ACZTSSe thin films, Fourier‐transform infrared (FTIR) characterizations were employed for P_2_S_5_, P_2_S_5_+CuCl, P_2_S_5_+Zn(CH_3_COO)_2_, and P_2_S_5_+SnCl_4_ 5H_2_O. As shown in **Figure** **2**, in the untouched P_2_S_5_ powder, a stretching vibrational mode peak at 926 cm^−1^ was detected, corresponding to the bond vibrations of P═S.^[^ ^39^, ^40^ ^]^ However, the peak of P═S shifts to a lower wavenumber of 916 cm^−1^ in the P_2_S_5_+CuCl mixture, indicative of a stronger interaction with CuCl. Meanwhile, the P═S stretching vibrational mode shifts to a lower wavenumber of 912 and 915 cm^−1^ in the P_2_S_5_+Zn(CH_3_COO)_2_ and P_2_S_5_+SnCl_4_ 5H_2_O samples, respectively. These shifts demonstrated the formation of strong coordination bonds between P_2_S_5_ and Cu^+^, Zn^2+^, and Sn^4+^ in ACZTS precursor solutions and ACZTS films, which is consistent with the calculated results.

To further verify the modulation effects of P_2_S_5_ in the crystallization of ACZTSSe films, the XRD patterns of all ACZTS precursor films are further collected from different P_2_S_5_ concentrations (Figure S3, Supporting Information), only diffraction peaks of kesterite structure are observed for all films with no secondary phase, and the half height width (FWHM) of ACZTS decreased gradually with the increase of P_2_S_5_ concentration. In particular, when the P_2_S_5_ concentration increased from 0 to 0.15 mg mL^−1^ (Figure S4, Supporting Information), the FWHM of ACZTS became sharply smaller, indicating that P_2_S_5_ has the effect of promoting crystal growth and improving crystalline quality. Figure S5a,b (Supporting Information) shows that only diffraction peaks for ACZTSSe in all P_2_S_5_ additive films, and the full‐width at half maximum of P_2_S_5_ additive samples is smaller, indicating that its crystallization is better. The Raman results are also shown in Figure S5c,d (Supporting Information), which demonstrate that all samples are composed of kesterite structure, which is consistent with the XRD results.^[^ ^16^, ^41^ ^]^

**Figure 3:**
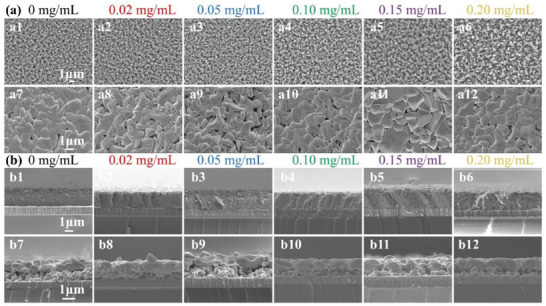
a) Top‐view SEM images of ACZTS precursor films and the final ACZTSSe absorbers under 0, 0.02, 0.05, 0.10, 0.15, and 0.20 mg mL^−1^ P_2_S_5_ concentrations. b) Cross‐section SEM images of ACZTS precursor films and the final ACZTSSe absorbers under 0, 0.02, 0.05, 0.10, 0.15, and 0.20 mg mL^−1^ P_2_S_5_ concentrations.

The surface and cross‐sectional morphologies of the ACZTSSe films with different concentrations of P_2_S_5_ or without P_2_S_5_ additive were studied by scanning electron microscopy (SEM) characterization. It is clearly demonstrated that P_2_S_5_ has an important effect on the surface morphology of the ACZTSSe thin film (**Figure** **3**a), the top‐view images of the films are well crystallized and covered with large grains with the increased P_2_S_5_ concentrations. Cross‐view morphologies in Figure 3b clearly indicate that all the ACZTSSe films exhibit a bilayer structure consisting of top and bottom grains, and the top grains are notably larger than those on the bottom. Finally, the upper layer grains under the 0.15 mg/mL P_2_S_5_ concentration absorber are larger than other samples, indicating improved crystallinity, which consists of XRD results. Furthermore, the reason for the changes in grain structure could be attributed to the addition of P_2_S_5_, which leads to large‐size agglomeration in the precursor films, it is more beneficial to grain growth in the process of high temperature selenization. Figure S6 (Supporting Information) shows the schematic illustration of the growth mechanism for the P_2_S_5_ additive film during the selenization process. These results reveal that the grain growth rate of the ACZTSSe film with P_2_S_5_ additive is faster than without P_2_S_5_ additive film at the equal selenization conditions, and the P_2_S_5_ additive led to increased grain size and enhanced ACZTSSe crystallization.^[^ ^42^ ^]^

UV–vis spectrum was also used to further evaluate the optical characteristics of the ACZTSSe film (Figure S7, Supporting Information). The absorption intensities of P_2_S_5_ additive films are obviously enhanced in the light absorption range of 380–1060 nm, this will strengthen the utilization rate of thin film, which is conducive to the corresponding photovoltaic devices generating a larger light current density. The higher light absorption capability is mainly attributed to the large‐size nucleation and better crystallization process of ACZTSSe films with P_2_S_5_ additive.^[^ ^39^ ^]^

**Figure 4:**
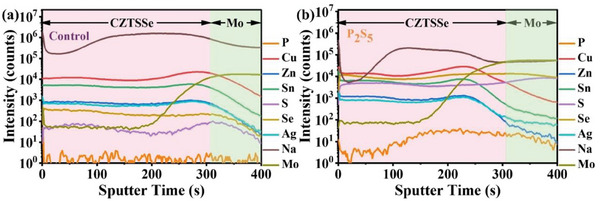
TOF‐SIMS elemental depth profiles of the a) control and b) P_2_S_5_ additive ACZTSSe absorbers.

Besides, the distribution of the elements within the selenized ACZTSSe absorber and P_2_S_5_ additive (0.15 mg/mL) absorber were carried out by time‐of‐flight secondary ion mass spectrometry (TOF‐SIMS) with two modes of positive and negative for cation elements and anionic elements, respectively, as shown in **Figure** **4**. It can be clearly seen that Cu, Zn, Sn, and Ag are uniformly distributed in two kinds of prepared samples. Moreover, P element is mainly present in the absorber, indicating that P_2_S_5_ additive really can improve crystal growth, which is consistent with the SEM results.^[^ ^43^, ^44^ ^]^

To investigate the valence states of various elements in the P_2_S_5_ additive in ACZTSSe absorbers, the XPS characterization method was used to detect these thin films. The XPS spectra of the key elements in the P_2_S_5_ additive samples are shown in Figure S8 (Supporting Information). Figure S8a (Supporting Information) shows the comprehensive XPS spectrum, indicating the presence of C, Cu, Zn, Sn, Ag, S, Se, and P elements in the synthesized film. The XPS spectra of the elements Cu 2P, Zn 2P, Sn 3d, S 2P, Se 3d, Ag 3d, and P 2P and their fitted curves are represented in Figure S8b–h (Supporting Information). The C1s peak at ≈284.8 eV was used as the internal standard for calibrating the binding energy. The two distinct peaks of Cu 2p were approximately present at 951.8 and 932.0 eV with a split orbit of 19.8 eV were attributed to the Cu 2p_1/2_ and Cu 2p_3/2_, respectively, which was consistent with the previously reported oxidation state of Cu^+^.^[^ ^4^, ^45^ ^]^ Figure S8c (Supporting Information) shows that the Zn 2p in the XPS spectra at 1021.9 and 1044.9 eV were the Zn2p_3/2_ and Zn 2p_1/2_, indicating that Zn existed in the oxidation state of Zn^2+^.^[^ ^42^ ^]^ There were also two peaks that belong to Sn 3d in the XPS spectrum at 486.3 and 494.7 eV with a peak separation value of 8.4 eV, which was well consistent with Sn^4+^.^[^ ^33^ ^]^ The XPS spectrum of S2p of the ACZTSSe with P_2_S_5_ additive sample is displayed in Figure S8e (Supporting Information). Since the S 2p core energy level overlaps with the Se 3p core energy level, these binding energies at 159.8, 160.4, 161.1, and 166.1 eV were recognized based on the Gauss‐Lorentz fitting method, which were Se 2p_3/2_, S 2p_3/2_, S 2p_1/2_, and Se 2p_1/2_, respectively.^[^ ^46^ ^]^ The binding energies of 160.4 and 161.1 eV were S 2p_3/2_ and S 2p_1/2_ S, respectively. Se 3d was able to obtain two peaks at 54.0 eV and 54.9 eV (Figure S8f, Supporting Information), which were Se 3d_3/2_ and Se 3d_1/2_, respectively, corresponding with the valence state of Se in CZTSSe films.^[^ ^47^ ^]^ The peaks of 367.7 and 373.8 eV in Ag 3d were Ag 3d_5/2_ and Ag 3d_3/2,_ respectively.^[^ ^46^ ^]^ The XPS results revealed the chemical valence state of Ag (I) in the final films. Figure S8h (Supporting Information) observed that the P 2p XPS peaks at 133.7 eV and 138.2 eV were consistent with the P 2p_1/2_ and P 2p_3/2_, indicating that P existed in the oxidation state of P^5+^.^[^ ^48^, ^49^ ^]^ The peak splitting values and peak positions of all elements agree well with the CZTSSe films oxidation states, respectively.

**Figure 5:**
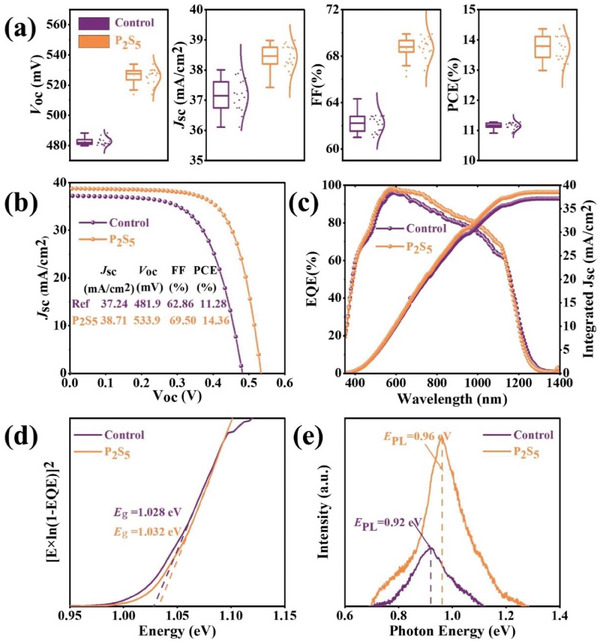
a) Distributions of *V*_OC_, *J*_SC_, FF, and PCE of control and 0.15 mg mL^−1^ P_2_S_5_ additive ACZTSSe devices. b) The *J–V* characteristics of the champion devices with and without P_2_S_5_ additive. c) EQE characterization and the respective integrated current densities with or without P_2_S_5_ additive. d) Bandgaps derived from the EQE spectra. e) Steady‐state PL spectra of ACZTSSe devices with and without P_2_S_5_ additive.

The improved crystallization quality of ACZTSSe by the P_2_S_5_ additive encouraged us to further study the consequence of the P_2_S_5_ additive on the final manufactured sample. The effect of P_2_S_5_ concentrations on two kinds of devices performance was summarized (**Figures** **5**a and S9a–d and Table S1, Supporting Information). Clearly, the optimal PCE was acquired at the concentration of 0.15 mg/mL, and the short‐circuit current density (*J*_SC_), open‐circuit voltage (*V*_OC_), and fill factor (FF) of this champion device are 38.71 mA cm^2^, 533.9 mV, and 69.50%, respectively, resulting in a power conversion efficiency (PCE) of 14.36% as shown in **Table** **1**. In addition, the device without P_2_S_5_ additive exhibited an average *J*_SC_ of 37.15 ± 0.52 mA cm^−2^, an average *V*_OC_ of 482.69 ± 2.39 mV, an average FF of 62.19 ± 0.81%, and an average PCE of 11.15 ± 0.12%. While the device based on the P_2_S_5_ additive presented an average *J*_SC_ of 38.44 ± 0.41 mA cm^−2^, an average *V*_OC_ of 525.97 ± 5.07 mV, an average FF of 68.64 ± 1.00%, and an average PCE of 13.74 ± 0.40%. It was clearly seen that all the modified photovoltaic parameters were improved via the P_2_S_5_ additive, due to improved ACZTSSe film quality owing to increased gain size and improved crystallinity, facilitated interfacial charge transfer, and reduced bulk defects of the ACZTSSe absorber, which resulted in reduced nonradiative recombination losses. The more detailed explanation will be given in the following sections.

**Table 1:** Detailed diode parameters of control and P_2_S_5_ additive champion devices are extracted using Site's methods from *J–V* curves.^[^ ^54^ ^]^ The values of *E*_g_ were derived from analysis of the EQE spectrum (Figure S6, Supporting Information).

Device	*J*_SC_ [mA cm^−2^]	*V*_OC_ [mV]	FF [%]	PCE [%]	*A*	*J*_0_ [mA cm^−2^]	*R*_s_ [Ω cm^2^]	*R*_sh_ [Ω cm^2^]	*G*_sh_ [mS cm^−2^]	*E*_g_ [eV]	*V*_OC_ deficit [V]
Control	37.24	481.99	62.86	11.28	2.10	4.1 × 10^−3^	0.72	1266	0.79	1.028	0.3099
P_2_S_5_	38.71	533.89	69.50	14.36	1.67	1.3 × 10^−4^	0.57	1370	0.73	1.032	0.2617

*J*–*V* curves and external quantum efficiency (EQE) spectra of the champion control and P_2_S_5_ additive devices are illustrated in Figures 5b,c and S10 (Supporting Information). Relatively higher *V*_OC_, *J*_SC_, and FF of the P_2_S_5_ additive samples are also in exact agreement with its relatively larger shunt resistance (*R*_sh_), lower series resistance (*R*_s_), and much lower reverse saturation current (*J*_0_) (Table 1 and Figure S11, Supporting Information).^[^ ^24^, ^43^ ^]^ By integrating the area of the EQE curves, the calculated *J*_SC_ agrees well with the *J*_SC_ values extracted from the *J*–*V* curves (Figure 5c). Furthermore, the external quantum efficiency (EQE) data demonstrates that the P_2_S_5_ additive device has a better photoresponse at long wavelengths (Figure 5c), illustrating less nonradiative recombination in the ACZTSSe absorber.^[^ ^4^ ^]^ The improvement of the EQE response of the P_2_S_5_ additive device at the long wavelength region is as desired, because due to the improvement of the crystallization quality, the grain boundaries along the collection paths are fewer, which can enhance the probability of optical carrier collection.^[^ ^40^ ^]^ Besides, it can be seen from the results in Table 1, Figures 5d and S12 (Supporting Information), the optical bandgap (*E*_g_) of the device with P_2_S_5_ additive and without P_2_S_5_ additive is 1.032 and 1.028 eV, and the Urbach energy (*E*_u_) is 27.96 and 28.63 meV, revealing that the bandgaps and *E*_u_ of the two devices are almost unchanged, which demonstrates that the increase of photovoltaic parameters are mainly due to the P_2_S_5_ additive. The *J*_SC_ values for the control and P_2_S_5_ devices, as determined by EQE characterization, are 37.16 mA cm^2^ and 38.54 mA cm^2^, respectively. These values are in close agreement with those derived from the *J*–*V* measurements. The minor discrepancy in *J*_SC_ values from EQE is attributed to the use of monochromatic light in the EQE testing process. The higher *J*_SC_ observed in the P_2_S_5_ additive CZTSSe solar cell is a contributing factor to the improved device performance. The detailed performance parameters of the CZTSSe solar cells reported in recent years are summarized in Table S2 (Supporting Information), which shows that the *V*_OC_/*V*_OC_^SQ^ (67.2%) and *V*_OC,def_ (0.2617 V) are lower than the other devices through the P_2_S_5_ additive. Figure 5e shows the photoluminescence (PL) spectroscopy emission peak energy (*E*_PL_) of Control and P_2_S_5_ additive device is 0.96 and 0.92 eV, respectively. The *E*_PL_ of Control and P_2_S_5_ additive device is much lower than the corresponding *E*_g_, suggesting that band‐to‐impurity transition is dominant in control and P_2_S_5_ additive device.^[^ ^50^ ^]^ In brief, both control and P_2_S_5_ additive device have serious band tailing effects. The band‐edge energy and bandgap fluctuations caused by Sn‐related defects such as [2Cu_Zn_ + Sn_Zn_] were considered to act as dominant recombination centers. The more result of the discussion will be given in the DLTS characterization technology section. In addition, the PL intensity of P_2_S_5_ additive device is significantly increased compared to control device, possibly indicating lower nonradiative recombination of P_2_S_5_ additive, which could further reduce the *V*_OC_‐deficit of CZTSSe solar cells.

For further research the effects of P_2_S_5_ additive on the photoelectric performance of ACZTSSe solar cells. Figure S13a (Supporting Information) provides the capacitance–voltage (*C*–*V*) curves of the control device and the P_2_S_5_ additive device, and the detailed corresponding carrier concentration (*N*_C–V_), depletion width (*W*_d_), and built‐in voltage (*V*_bi_) extracted from the *C*–*V* curves are the same as the previous reports.^[^ ^50^, ^51^, ^52^ ^]^ The P_2_S_5_ additive device exhibits a boosted depletion width. In fact, a wider depletion region is more beneficial for the separation of photogenerated carrier, particularly in the long wavelength range, which corresponds well with the EQE measurement shown in Figure 5c. Moreover, the *V*_bi_ of the two photovoltaic devices are 616 mV, and 776 mV without and with P_2_S_5_ additive cells, respectively (Figure S13b, Supporting Information). It is widely recognized that a heightened *V*_bi_ contributes supplementary momentum for charge carrier separation, transport, and extraction, thereby effectively preventing the recombination of electron–hole pairs in kesterite devices.^[^ ^53^ ^]^ Figure S13c (Supporting Information) shows a much lower carrier concentration (*N*_CV_) of 5.64 × 10^16^ and 2.61 × 10^16^ cm^−3^ for control and P_2_S_5_ additive devices, respectively. Based on the above measurements, the P_2_S_5_ additive strategy is more beneficial to improve the device performance.

**Figure 6:**
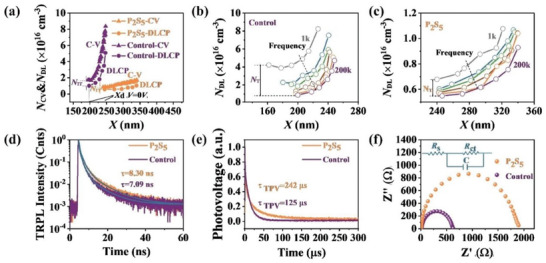
a) Drive‐level capacitance profiling (DLCP) and *C–V* profiles of the control sample and P_2_S_5_ additive sample. *N*_DL_ of b) Control device and c) P_2_S_5_ additive device measured at frequencies from 1 to 200 kHz. d) TRPL decay curves of ACZTSSe devices with and without P_2_S_5_ additive. e) TPV spectra of ACZTSSe devices with and without P_2_S_5_ additive. f) Nyquist plots of the devices with and without P_2_S_5_ additive.

To further explore the mechanism of the carrier density and collection properties of the ACZTSSe absorbers, the *C*–*V* profile and drive‐level capacity profile (DLCP) are performed in **Figure** **6**a–c. Likewise, a larger *X*_d_ is observed in the P_2_S_5_ additive devices, which is consistent with the *C*–*V* results in Figure S13c (Supporting Information). It has been well known that *C*‐*V* results (*N*_CV_) include the effect of free carrier density, bulk defect density and interface defect density. Conversely, the DLCP results (*N*_DL_) only account for free carrier density and bulk defect density. Therefore, the lower value of *N*_C‐V_–*N*_DL_ (zero bias) indicates a lower interface traps (*N*_IT_). Also, defects are incapable of significant response at high frequencies, allowing only the free carriers to dictate the DLCP signal. However, when it comes to low frequencies, the capacitance value is a representation of the response from all free carriers and deep traps, showing that DLCP can identify their reactions at this frequency level.^[^ ^24^, ^50^ ^]^ Consequently, the distinction in DLCP signals between high and low frequencies is predominantly a reflection of the bulk defect density. Meanwhile, the values of *N*_CV_ and *N*_DL_ can be calculated according to the following equations:^[^ ^24^, ^40^ ^]^

(1) $$X_{d}=\frac{A\varepsilon_{0}\varepsilon }{C}$$

(2) $$\frac{1}{C^{2}}=\frac{2\left(V_{\mathrm{bi}}-V\right)}{qA^{2}\varepsilon_{0}\varepsilon N_{\mathrm{cv}}}$$

(3) $$N_{\mathrm{cv}}=\frac{C^{3}}{qA^{2}\varepsilon_{0}\varepsilon }{\left(\frac{dC}{dV}\right)}^{-1}$$

(4) $$N_{\mathrm{DL}}=-\frac{1}{2q\varepsilon_{0}\varepsilon A^{2}}\frac{C_{0}^{3}}{C_{1}}$$

where *A* is the total area of the solar cells (*A* = 0.2304 cm^2^), 𝑞 is the electron charge, 𝜀_0_ is the vacuum permittivity, 𝜀 is the relative dielectric constant of CZTSSe, and 𝐶_0_ and 𝐶_1_ are two quadratic fitting parameters. The corresponding results were shown in **Table** **2**. And more encouragingly, the values of *N*_CV_, *N*_DL_, *N*_IT_, and *N*_T_ are further reduced for P_2_S_5_ additive devices, they are from 1.73 × 10^16^ to 0.85 × 10^16^ cm^−3^, 1.02 × 10^16^ to 0.57 × 10^16^ cm^−3^, 0.71 × 10^16^ to 0.28 × 10^16^ cm^−3^ and 3.34 × 10^16^ to 0.13 × 10^16^ cm^−3^ for control and P_2_S_5_ additive devices, respectively. Such results again corroborate that the P_2_S_5_ additive is more favorable for carrier collection and separation and has effectively reduced the bulk defect density.

**Table 2:** Summary of the results derived from *C*–*V* and drive‐level capacitance profiling (DLCP) measurements.

Device	*X*_d_ [µm]	*N*_CV_ [cm^−3^]	*N*_DL_ [cm^−3^]	*N*_IT_ [cm^−3^]	*N*_T_ [cm^−3^]
Control	0.195	1.73 × 10^16^	1.02 × 10^16^	0.71 × 10^16^	3.34 × 10^16^
P_2_S_5_	0.244	0.85 × 10^16^	0.57 × 10^16^	0.28 × 10^16^	0.13 × 10^16^

To study the charge carrier dynamics of the CZTSSe devices with and without P_2_S_5_ additive, time‐resolved photoluminescence (TRPL) measurements and transient photovoltage decay (TPV) spectroscopy are performed in Figure 6d,e. The obtained TRPL decay curves were fitted using a biexponential decay equation: *τ*_avg_ = (*A*_1_ *τ*_1_^2^ + *A*_2_ *τ*_2_^2^) / (*A*_1_ *τ*_1_ + *A*_2_ *τ*_2_), where *A*_1_ and *A*_2_ are decay amplitudes, *τ*_1_ is the faster decay component resulting from bimolecular recombination, and *τ*_2_ is the slower decay component caused by defect‐assisted recombination. A summary of the TRPL fitting results can be found in Table S3 (Supporting Information) for detailed examination. Clearly, the P_2_S_5_ additive films exhibit an extended average lifetime of 8.30 ns, in stark contrast to the 7.09 ns observed in the control films (Figure 6d), indicating reduced nonradiative recombination centers of P_2_S_5_ additive films.^[^ ^24^ ^]^ In addition, the decay time of photovoltage in ACZTSSe devices with P_2_S_5_ additive (242 µs) is longer than that of the control devices (125 µs) by TPV measurement (Figure 6e), indicating the slower recombination of photogenerated charge carriers.^[^ ^33^, ^42^ ^]^

Next, the electrochemical impedance spectroscopy (EIS) response of the most exemplary samples was explored to better understand the dynamics of carrier recombination and transport within the devices (Figures 6f and S14, and Table S4, Supporting Information).

By applying a refined Randles equivalent circuit, the semicircle in the EIS measurements are correlated with a composite of the charge transport resistance (*R*_s_) and the recombination resistance (*R*_ct_). Figure 6f illustrated that devices with the P_2_S_5_ additive exhibit a lower *R*_s_ and a higher *R*_ct_ compared to the control devices, indicating enhanced charge transport and suppressed nonradiative carrier recombination with the P_2_S_5_ additive.^[^ ^42^ ^]^

**Figure 7:**
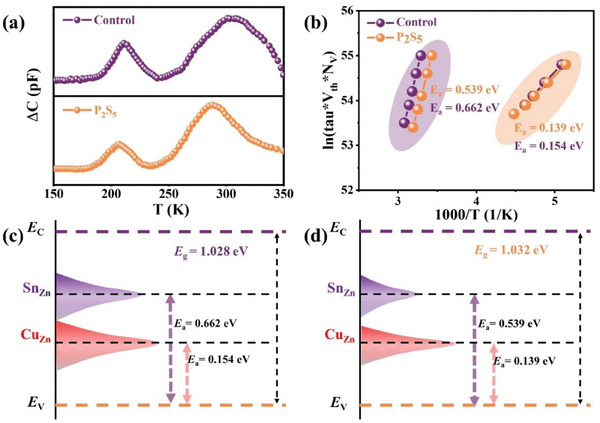
a) C‐DLTS spectra and b) Arrhenius plots of the control device and the P_2_S_5_ additive device. The evolution of variation in major defects of c) the control device and d) the P_2_S_5_ additive device.

Finally, capacitance‐mode deep level transient spectroscopy (*C*‐DLTS) was utilized to investigate the deep‐level defect properties of the bulk CZTSSe films in the champion devices. **Figure** **7**a demonstrates the DLTS signals under reverse bias (*U*_R_) was 0.30 V, the pulse voltage (*U*_P_) was −0.40 V, and the pulse width (*t*_P_) was 10 ms of the control and the P_2_S_5_ additive champion devices. Apparently, only one peak is observed for all the devices. Furthermore, the activation energy (*E*_a_) and the integrated density (*N*_T_) of the defects for the two devices were obtained from the Arrhenius plots shown in Figure 7b and listed in **Table** **3**, with further details provided in Figure 7c,d. The defects with lower *E*_a_ of 0.154 and 0.139 eV were assigned to Cu_Zn_ antisite acceptor defects, and those with higher *E*_a_ of 0.662 eV and 0.539 eV were Sn_Zn_ defects.^[^ ^34^, ^55^ ^]^ However, these defects are all “killer centers.”^[^ ^56^ ^]^ In recent years, considerable theoretical and experimental studies have demonstrated that Sn_Zn_ defects and their defect clusters ([2Cu_Zn_ + Sn_Zn_]) act as dominant nonradiative recombination center in kesterite devices, limiting the minority carrier lifetime and the *V*_OC_, and hindering the further improvement of the efficiency of photovoltaic devices.^[^ ^57^, ^58^ ^]^ It can be found that the *N*_T_ (2.99 × 10^11^ cm^−3^) of Cu_Zn_ defects and the *N*_T_ (2.59 × 10^13^ cm^−3^) of Sn_Zn_ defects are all decreased with the P_2_S_5_ additive treatment compared to the control device. Therefore, as calculated in theory, we believe that this defect suppression may be due to the ability of the P_2_S_5_ additive introduced in the precursor solution to influence the coordination environment of the cations in CZTSSe, especially during the high‐temperature selenization crystallization process through strong coordination with Zn^2+^ to stabilize the Zn sites, which can stabilize the CZTSSe lattice structure, reducing the concentration of Cu_Zn_ and Sn_Zn_ defects and it is also beneficial for the improvement of the *V*_OC_ deficit. These results demonstrated that the synergistic effect of the P_2_S_5_ additive strategy effectively suppresses the trapping defects in the depletion region of devices, which is advantageous for alleviating the serious band tail effect and bulk recombination, thereby boosting *V*_OC_ and FF in CZTSSe devices.

**Table 3:** Summary of the parameters of photoelectric properties from the *C*–*V* curves and C‐DLTS spectra of the control device and the P_2_S_5_ additive device.

Device	Apparent carrier concentration *N*_C–V_ [cm^−3^]	Depletion width *W*_d_ [µm]	Built‐in potential *V*_bi_ [mV]	Peaks temp. *T* [K]	Activation energy *E*_a_ [eV]	Defect densities *N*_T_ [cm^−3^]	σ_T_ [cm^2^]	Possible defect level
Control	5.64 × 10^16^	0.23	616	211	0.154	5.44 × 10^11^	1.45 × 10^−20^	Cu_Zn_
				304	0.662	3.07 × 10^14^	1.15 × 10^−13^	Sn_Zn_
P_2_S_5_	2.61 × 10^16^	0.29	776	206	0.139	2.99 × 10^11^	6.73 × 10^−21^	Cu_Zn_
				289	0.539	2.59 × 10^13^	2.90 × 10^−15^	Sn_Zn_

## 3 Conclusion

In conclusion, a new synergetic modulation and passivation strategy by regulating the metal cation coordination structure via P_2_S_5_ as an anion‐coordinate precursor additive is proposed. Theoretical calculations and the results of FTIR demonstrated that the addition of P_2_S_5_ has strong interaction with metal ions, especially for Zn^2+^, which can stabilize the kesterite lattice structure. Besides, the fabricated kesterite films have a longer lifetime. In addition, the *C*‐DLTS spectra demonstrated that the Cu_Zn_ and Sn_Zn_ defects are suppressed, significantly reducing the formation of non‐radiative recombination centers. As a result, the device with the P_2_S_5_ additive achieves the champion PCE of 14.36%. All the results show that P_2_S_5_ is a promising and novel anion‐coordinate precursor additive for obtaining superior quality kesterite films, advancing the photoelectric performance of kesterite solar cells.

## Conflict of Interest

The authors declare no conflict of interest.

## Supporting information

Supporting Information

## Data Availability

The data that support the findings of this study are available from the corresponding author upon reasonable request.
