# Supplementary material for: Synergistic Crystallization Modulation and Defects Passivation in Kesterite via Anion‐Coordinate Precursor Engineering for Efficient Solar Cells
Source: Adv Sci (Weinh). 2024 Jul 19;11(35):2405016. doi: 10.1002/advs.202405016 (PMC11425231; doi:10.1002/advs.202405016)
Supplement: Supplementary file 1 — Supporting Information [file ADVS-11-2405016-s001.docx]

Supporting Information

**Synergistic Crystallization Modulation and Defects Passivation in Kesterite via Anion-Coordinate Precursor Engineering for Efficient Solar Cells**

*Lijing Wang, Liangli Chu, Zhengji Zhou**^*^, Wenhui Zhou, Dongxing Kou, Yuena Meng, Yafang Qi, Shengjie Yuan, Litao Han, Gang Yang^*^, Zhuhua Zhang, Zhi Zheng^*^ and Sixin Wu^*^*

L. Wang, L. Chu, Z. Zhou, W. Zhou, D. Kou, Y. Meng, Y. Qi, S. Yuan, L. Han, S. Wu

Key Lab for Special Functional Materials, Ministry of Education, National and Local Joint Engineering Research Center for High-Efficiency Display and Lighting Technology, and School of Materials, Henan University, Kaifeng 475004, China

E-mail: [zzj@henu.edu.cn](mailto:zzj@henu.edu.cn); [wusixin@henu.edu.cn](mailto:wusixin@henu.edu.cn)

L. Wang, G. Yang

College of Physics and Electronic Engineering, Nanyang Normal University, Nanyang 473061, China

E-mail: 20171022@nynu.edu.cn

L. Chu, Z. Zhang

State Key Laboratory of Mechanics and Control for Aerospace Structures, Key Laboratory for Intelligent Nano Materials and Devices of Ministry of Education, Institute for Frontier Science, Nanjing University of Aeronautics and Astronautics, Nanjing 210016, China

College of Physics, Nanjing University of Aeronautics and Astronautics, Nanjing 211106, China

Z. Zheng

Inst Surface Micro & Nano Mat, Coll Adv Mat & Energy, Key Lab Micronano Energy Storage & Convers Mat He, Xuchang University, Xuchang, Henan 461000, China.

E-mail: zzheng@xcu.edu.cn

Experimental Section

*Materials:* Zinc acetate dihydrate (Zn(CH_3_COO)_2_·2H_2_O, 99.99%), cuprous chloride (CuCl, 99.999%), stannic chloride (SnCl_4_·5H_2_O, 99.9%,), silver chloride (AgCl, 99.5%), 2-methoxyethanol (C_3_H_8_O_2_, 99.8%) and cadmium sulfate (CdSO_4_, 99%) were purchased from Aladdin Company. Phosphorus pentasulfide (P_2_S_5_, 99%, P≥27%) was purchased from Macklin. Thiourea (SC(NH_2_)_2_, 99%) was purchased from Alfa Aesar Chemical Co. Ammonium hydroxide (NH_3_·H_2_O, 25%) was obtained from Beijing Chemical Works. Selenium particles (99.99%) were purchased from ZhongNuo Advanced Material Technology Co., Ltd. All chemicals and solvents were commercially available and used as received without further purification.

*Film preparation:* The (Ag,Cu)_2_ZnSnS_4_ (ACZTS) precursor solutions were prepared as follows: CuCl, Zn(CH_3_COO)_2_·2H_2_O, SnCl_4_·5H_2_O, AgCl, and thiourea were dissolved in the 2-methoxyethanol solvent (EGME) and stirred at 60 °C for an hour to obtain a colourless solution, then AgCl was dissolved in the solution for another an hour to get a final colourless solution (EGME-air precursor). The molar ratio of Ag / (Ag + Cu), (Ag + Cu) / (Zn + Sn), Zn/Sn and thiourea/metal are 0.05, 0.75, 1.18 and 2.05, respectively, and the concentrations of metal elements and thiourea are 0.65 mol l^-1^ and 1.33 mol l^-1^, respectively. For the P_2_S_5_ additive ACZTS precursor solutions, when AgCl was completely dissolved, the P_2_S_5_ of 0.02, 0.05, 0.10, 0.15 and 0.20 mg/mL concentrations was added to the solutions and stirred at 60 °C for an hour to obtain colourless solutions. The precursor films were prepared by spin coating the precursor solutions on cleaned Mo substrates at a spin speed of 3,000 r.p.m. s^-1^ for 30 s, followed by annealing on a 280 °C hot plate in the air. The coating and annealing processes were repeated 11 times for the appropriate thickness (~1.5 μm) to obtain the final precursor films. Then, the prepared precursor films were annealed with N_2_ flow in a graphite box with selenium-contained in a rapid thermal processer (RTP) furnace.

*Device preparation:* The typical ACZTSSe solar cells were prepared with a structure of glass/Mo/ACZTSSe/CdS/i-ZnO/ITO/Ag. Firstly, CdS buffer layer (50 nm) was deposited onto the selenized ACZTSSe films by a chemical bath-deposited (CBD) method. Then 50 nm zinc oxide (i-ZnO) and 200 nm indium tin oxide (ITO) window layers were deposited by radio frequency (RF) magnetron sputtering. Finally, the Ag grid electrode was thermally evaporated through a metal shadow mask as a current collector. Notably, MgF_2_ anti-reflection layer was evaporated onto the champion solar cells. The device was divided into nine small standard cells with an effective area of 0.21 cm^2^.

*Film and device performance Characterization:* The structural properties of the ACZTSSe thin films were analyzed by Bruker AXS (D8 Advance) X-ray diffraction (XRD) patterns with Cu K_α_ as the radiation (λ_Kα_ =1.5406 Å), diffraction data was collected at 0.04° steps from 10° to 80° in 2θ. Raman spectra were characterized by using microscopic confocal Raman spectrometry (Renishaw inVia) with an excitation of 532 nm. Fourier transform infrared (Nicolet 360 FTIR) spectra were acquired in the scanning range of 600-4000 cm^-1^. The top-view and cross-section scanning electron microscopy (SEM) images of the final ACZTSSe absorbers were characterized with an energy dispersive X-ray analyzer (FEI, Nova Nano SEM 450/EDS). Depth profiling data was acquired by a TOF-SIMS 5-100 secondary-ion mass spectrometry (SIMS) system (polarity: positive and negative mode for cation elements and anionic elements, respectively). The surface element valence state of the ACZTSSe thin films was analyzed by X-Ray photoelectron spectroscopy (XPS) system (AXIS, SUPRA+). The current density-voltage (*J*-*V*) characteristics were measured by a solar simulator calibrated with Keithley 2400 Source Meter under standard lighting conditions (AM 1.5 illumination, 100 mW cm^-2^). The external quantum efficiency (EQE) spectra was measured using a Zolix SCS100 QE system installed with a 150-W xenon light source and a lock-in amplifier. The capacitance-voltage (*C*-*V*) curves, capacitance-mode deep level transient spectroscopy (*C*-DLTS) measurements, the *C*-*V* and drive level capacitance profile (DLCP), transient photovoltage (TPV) spectra, the photoluminescence (PL) spectroscopy, time-resolved photoluminescence (TRPL) spectroscopy and electrochemistry impedance spectroscopy (EIS), are all measured according to our work published previously.^[1, 2]^

*Theoretical calculation method:* The first-principles calculations were carried out using the Density Functional Theory (DFT) as implemented in the Vienna Ab initio Simulation Package (VASP),^[3, 4]^ utilizing projector-augmented-wave (PAW) pseudopotentials.^[5]^ All of the structures were relaxed using the Perdew−Burke−Ernzerhof (PBE) exchange-correlation functional of the generalized gradient approximation (GGA) method.^[6]^ An energy cutoff of 400 eV was used in the calculations. The vacuum spacing in a direction perpendicular to the plane of the structure was 20 A for the surfaces. The Brillouin zone integration was performed using 3x2x1 Monkhorst-Pack k-point sampling for a structure. Electron energies were considered self-consistent when the energy change was less than 10^-5^ eV. The geometry optimization was considered converged when the Hellmann−Feynman force change was less than 0.02 eV/Å.

**
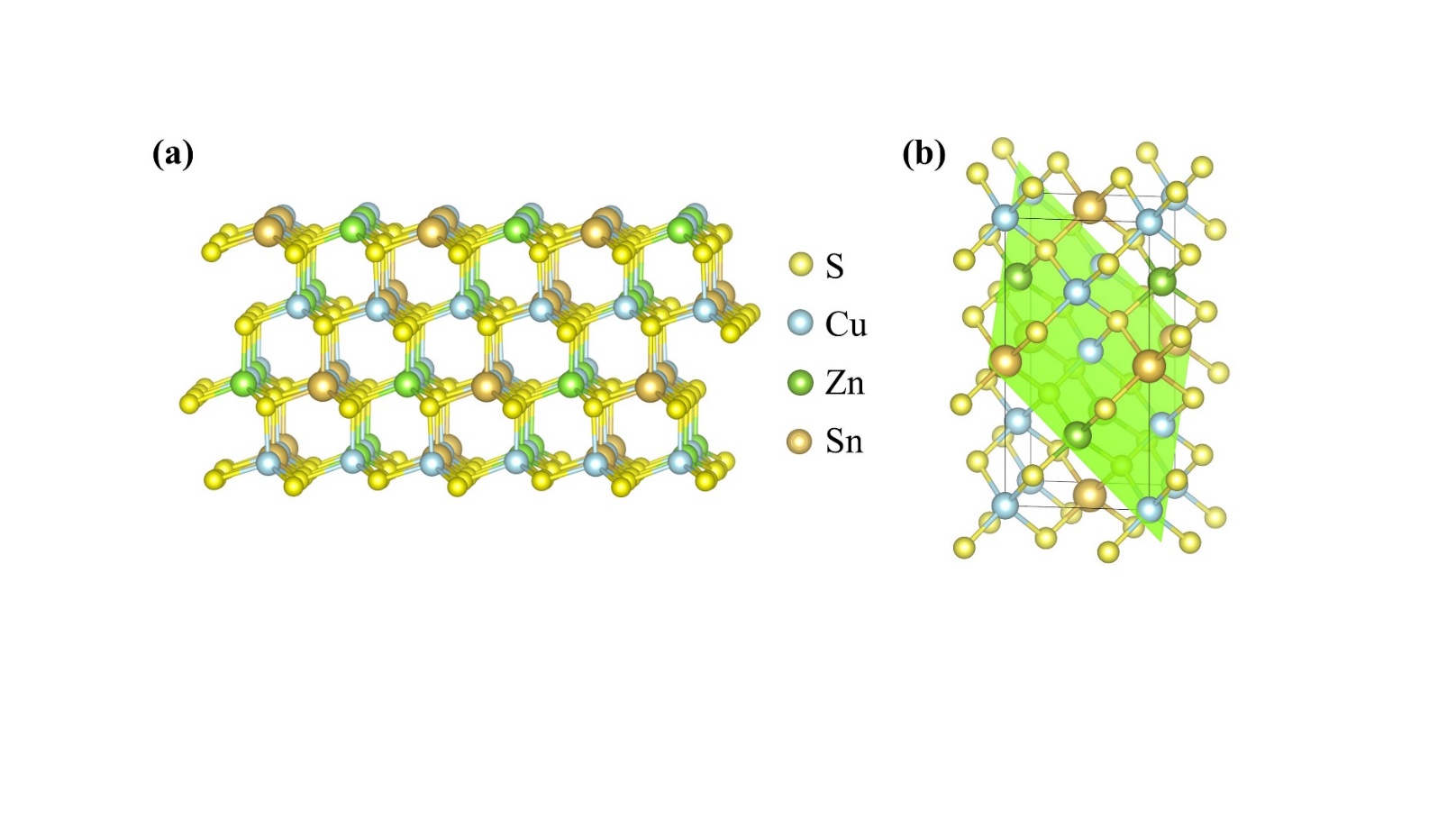
**

**Figure S1.** (a) The ideal crystal structure of kesterite CZTS. (b) (112) crystal plane of CZTS thin film.

**
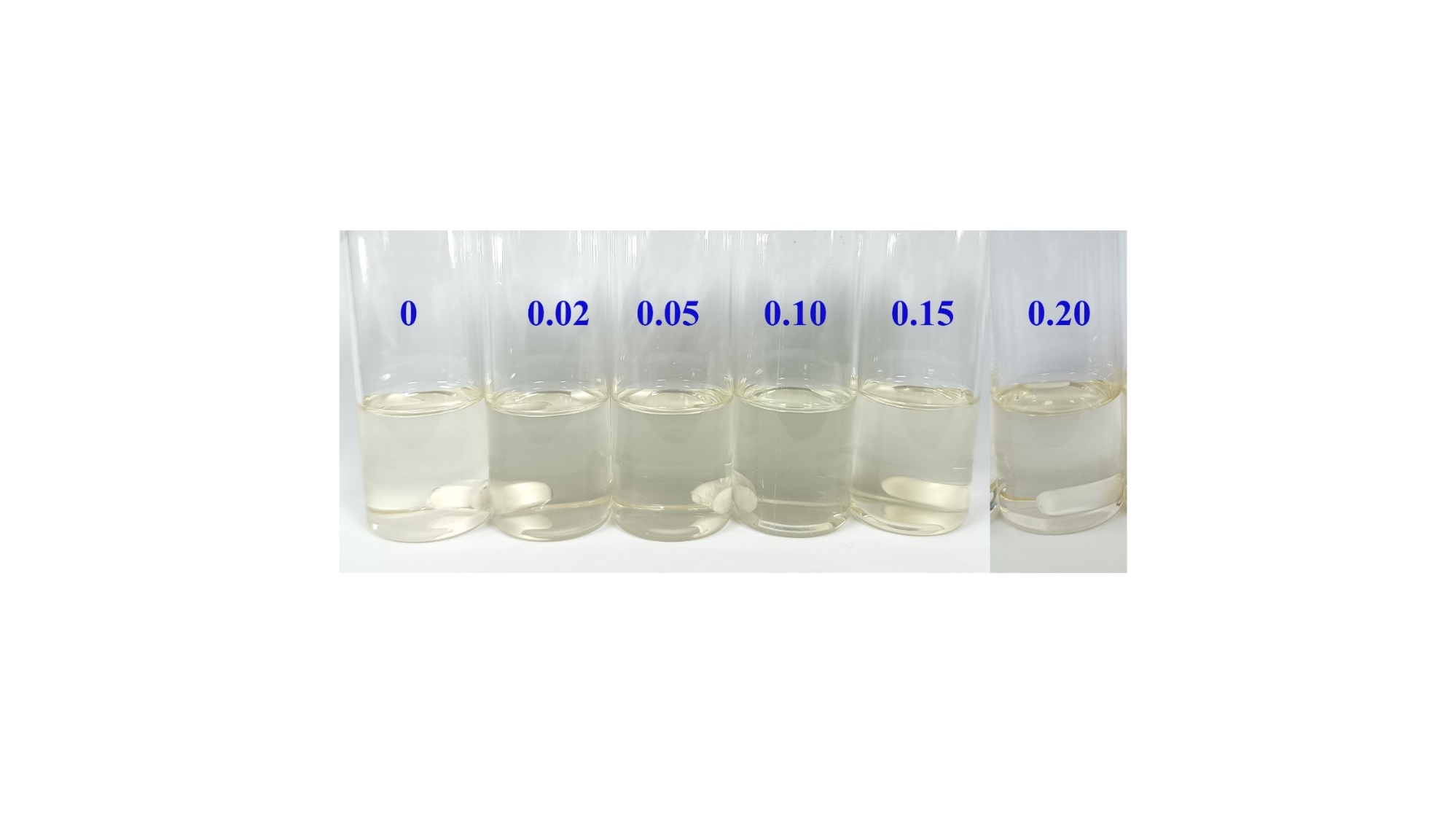
**

**Figure S2.** Digital photograph of a series of ACZTS precursor solutions prepared by dissolving CuCl, Zn(CH_3_COO)_2_ 2H_2_O, SnCl_4_ 5H_2_O, AgCl, SC(NH_2_)_2_ and P_2_S_5_ in 2-methoxyethanol with a concentration of P_2_S_5_ of 0, 0.02, 0.05, 0.1, 0.15, and 0.20 mg/mL, respectively.

**
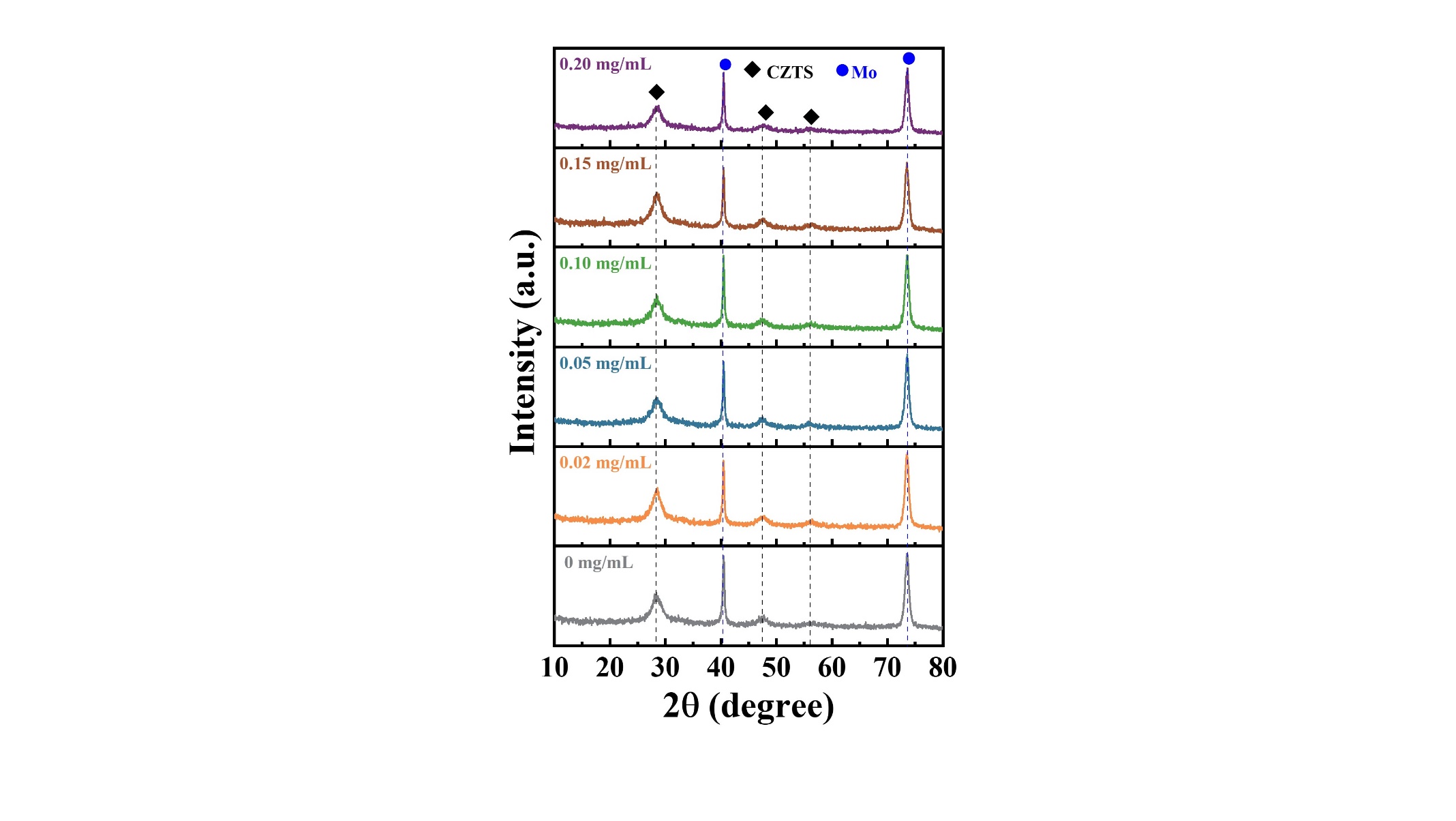
**

**Figure S3.** X-ray diffraction spectra of ACZTS precursor films with different P_2_S_5_ concentrations.


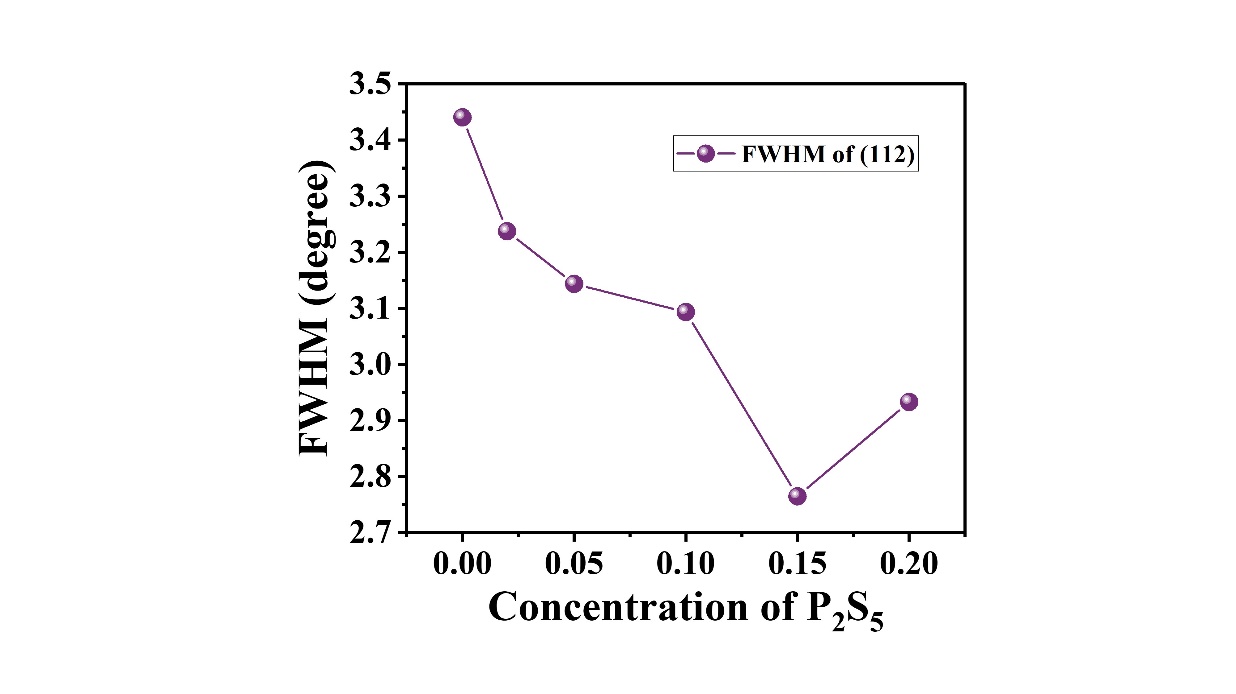


**Figure S4.** The FWHM of the ACZTS precursor films of (112) diffraction peak varies with different P_2_S_5_ concentrations.


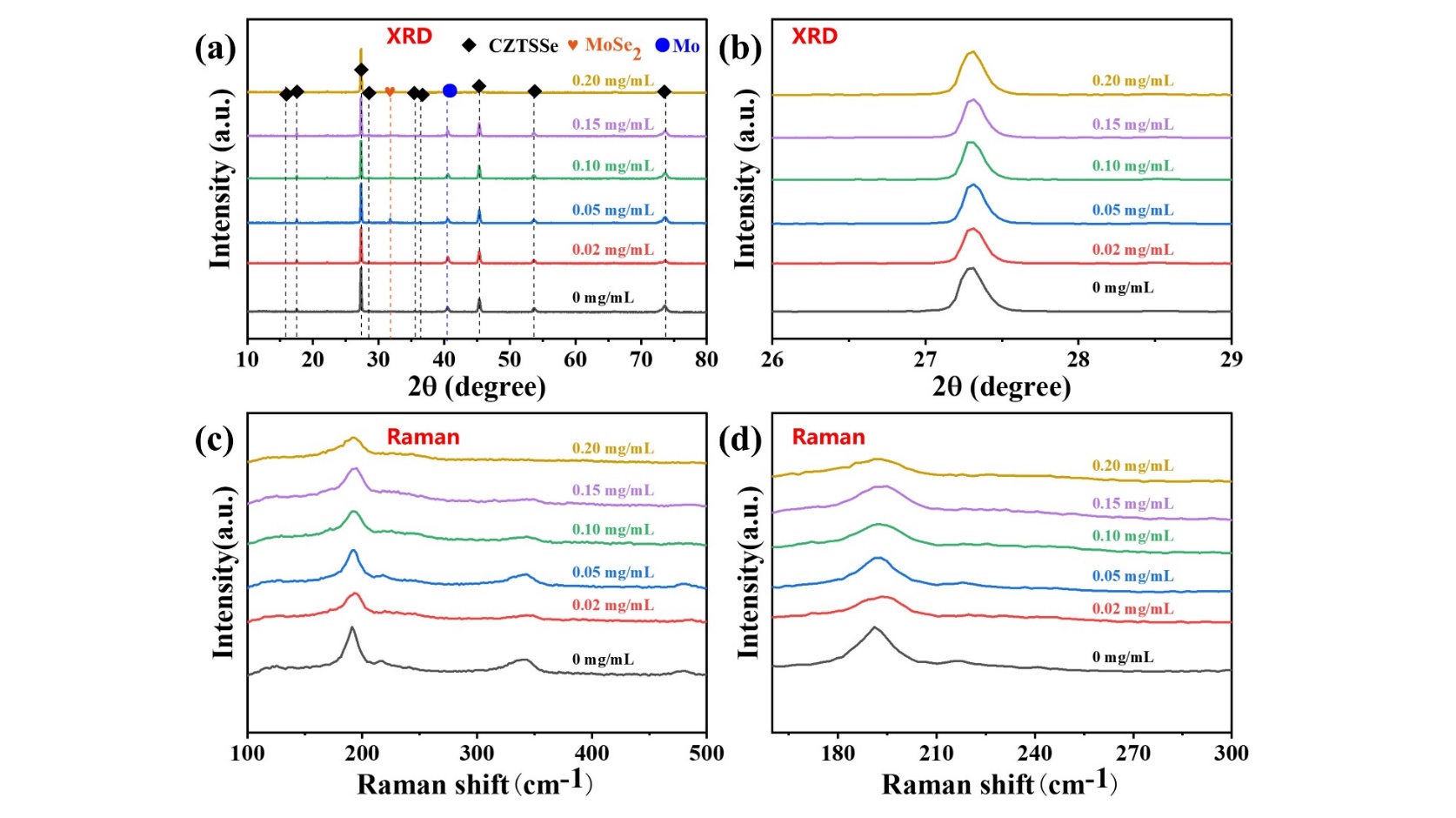


**Figure S5.** (a) X-ray diffraction spectra of ACZTSSe selenized films with different P_2_S_5_ concentrations. (b) Enlarged view of (112) peaks. (c) Raman spectra of ACZTSSe selenized films with different P_2_S_5_ concentrations. (d) Enlarged view of main kesterite type structure peaks.


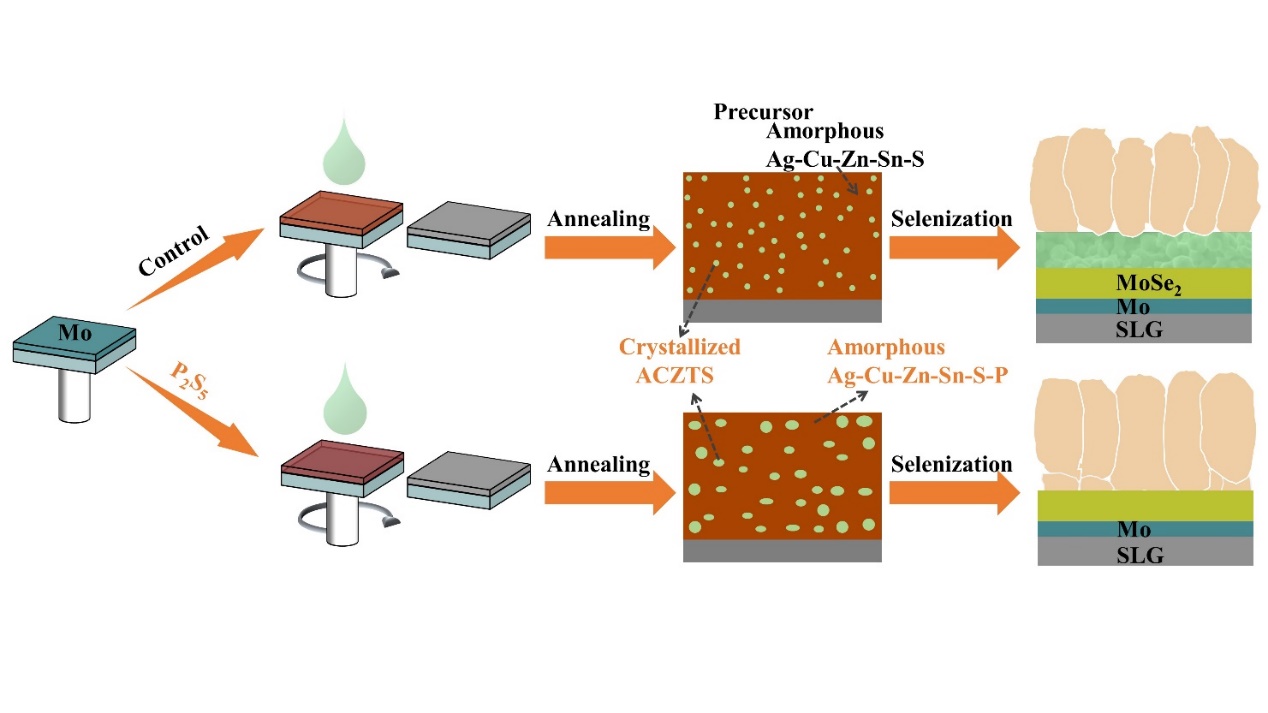


**Figure S6.** Schematic illustration of the growth mechanism for the P_2_S_5_ additive film during the selenization process.


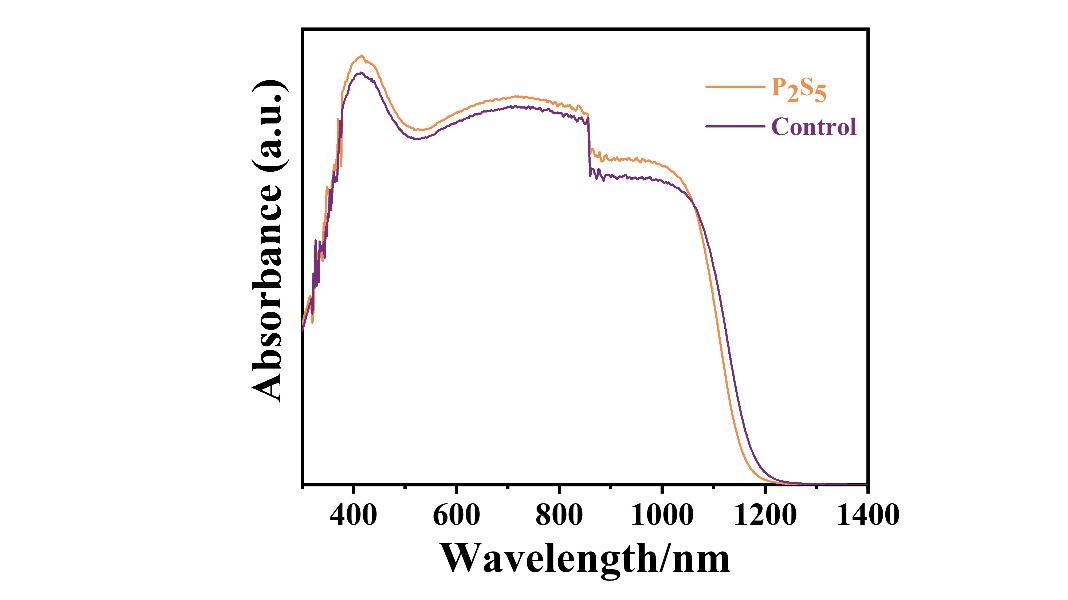


**Figure S7.** UV-vis absorption spectra of ACZTSSe thin films with and without P_2_S_5_ additive.


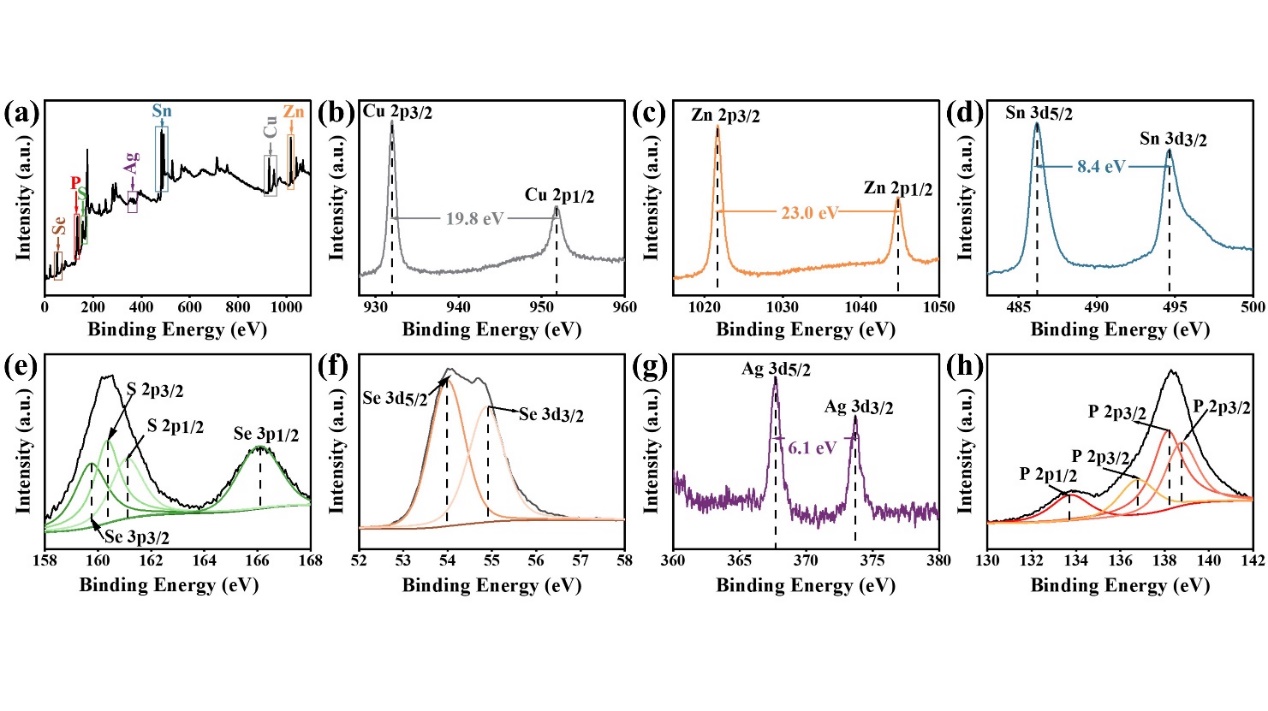


**Figure S8.** XPS spectra of ACZTSSe absorbers doped with 0.15 mg/mL P_2_S_5_ concentrations. (a) The full-scan spectrum, (b) Cu 2P, (c) Zn 2P, (d) Sn 3d, (e) S 2P, (f) Se 3d, (g) Ag 3d and (h) P 2P.


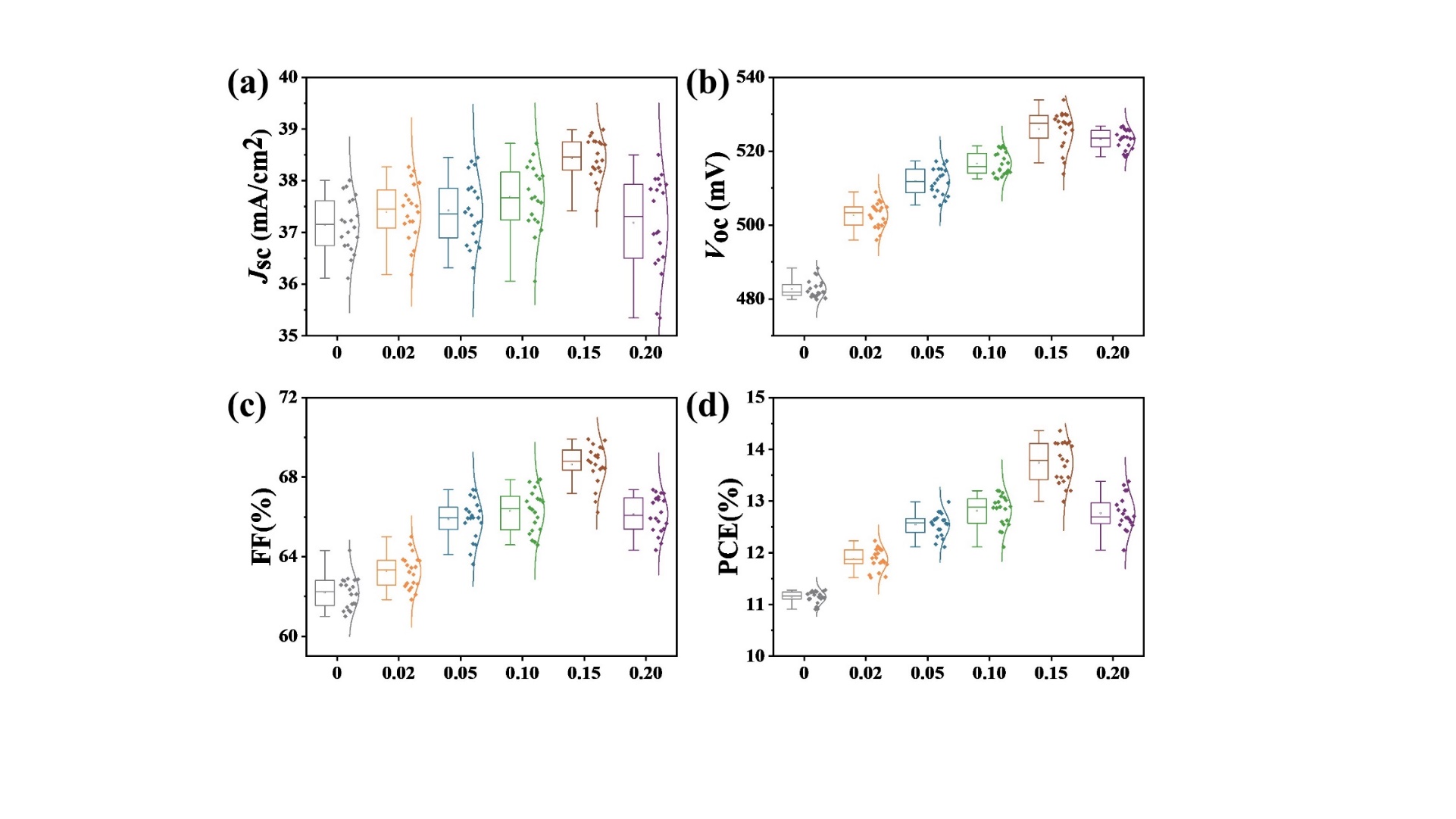


**Figure S9.** Device properties of ACZTSSe solar cells doped with 0, 0.02, 0.05, 0.10, 0.15, and 0.20 mg/mL P_2_S_5_ concentrations. Statistical distributions for (a) *J*_sc_, (b) *V*_oc_, (c) FF, and (d) PCE.


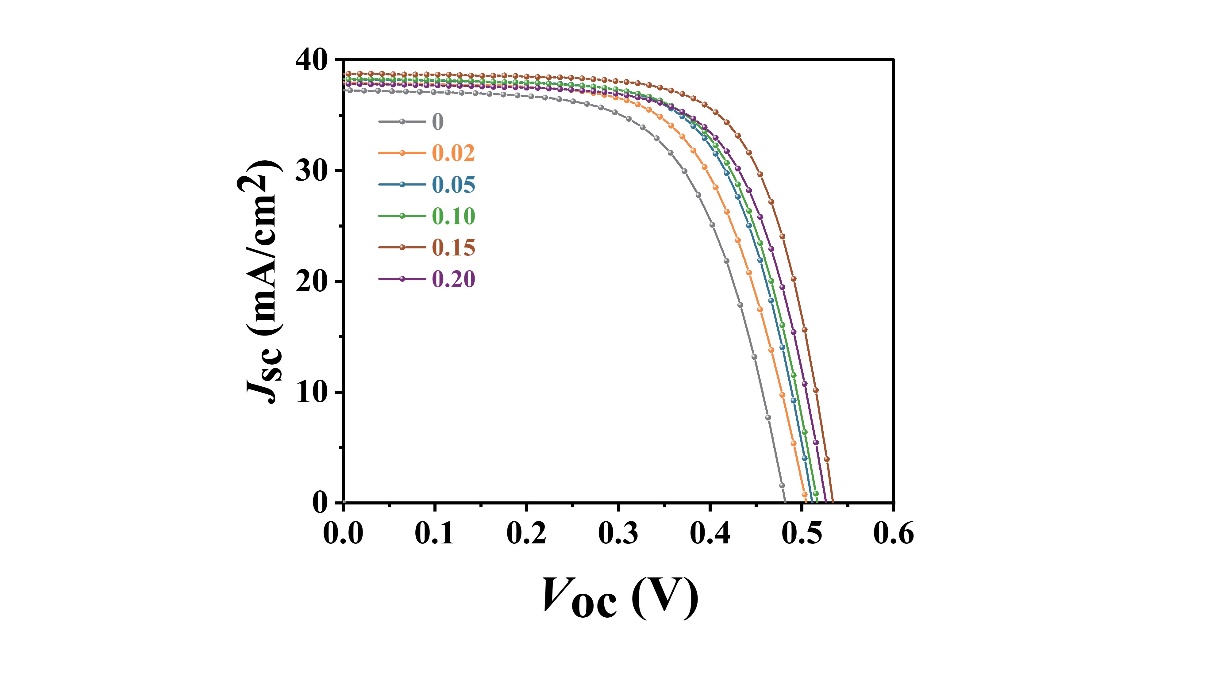


**Figure S10.** The *J*-*V* curves of ACZTSSe solar cells doped with 0, 0.02, 0.05, 0.10, 0.15, and 0.20 mg/mL P_2_S_5_ concentrations.


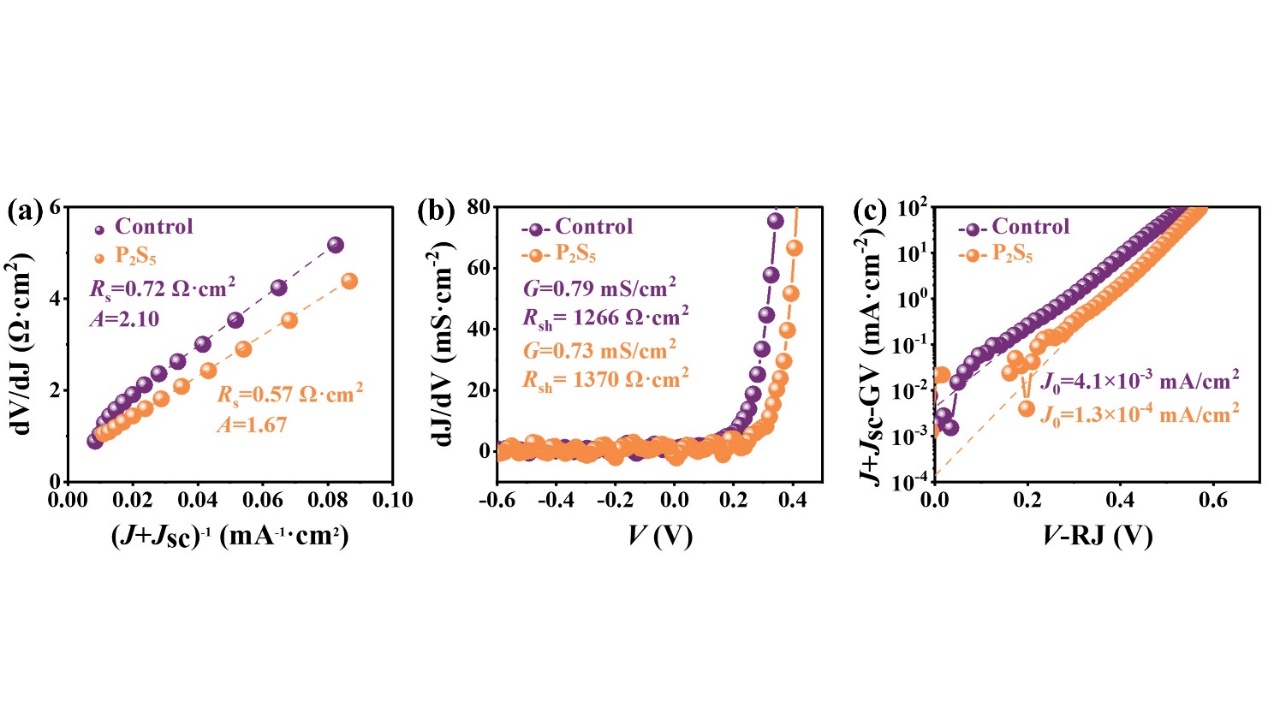


**Figure S11.** (a) Series resistance *R*_s_ and diode ideality factor A, (b) shunt resistance *R*sh and *G*, (c) reverse saturation current density *J*0 derived from the *J*-*V* curves of the Control and P_2_S_5_ additive champion devices according to the site’s method.^[7]^


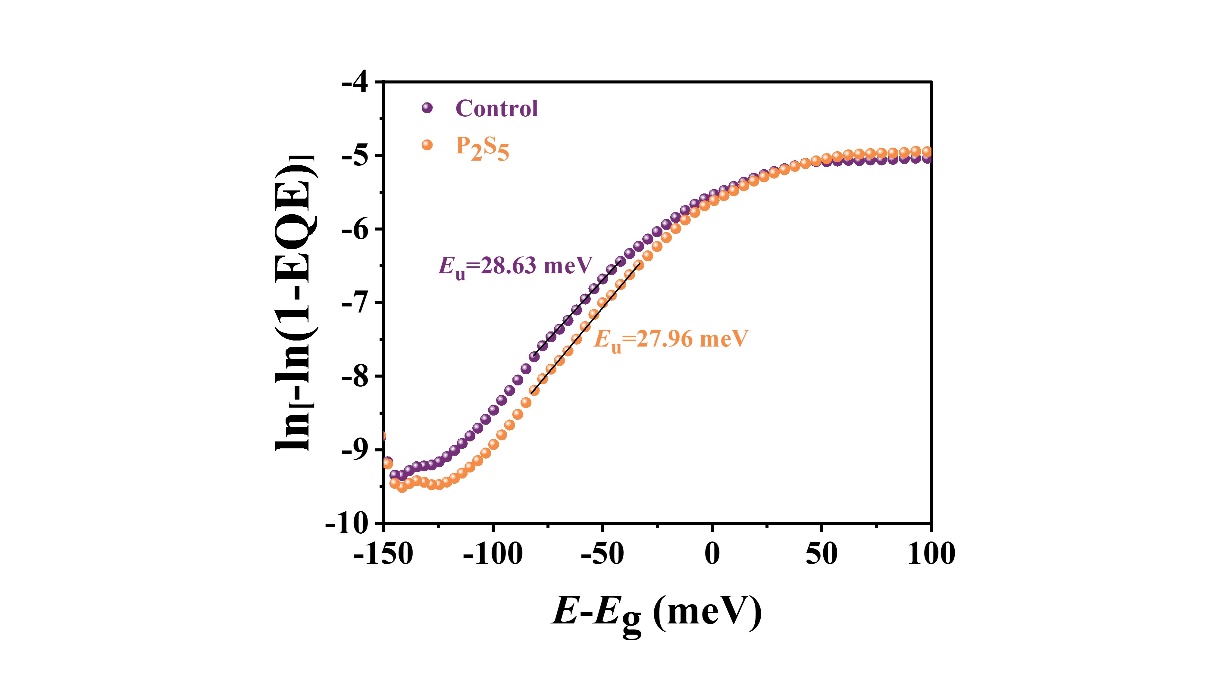


**Figure S12.** The extraction of the Urbach energy derived from ln(-ln(1-EQE)) vs. *E*-*E*_g_ plots.


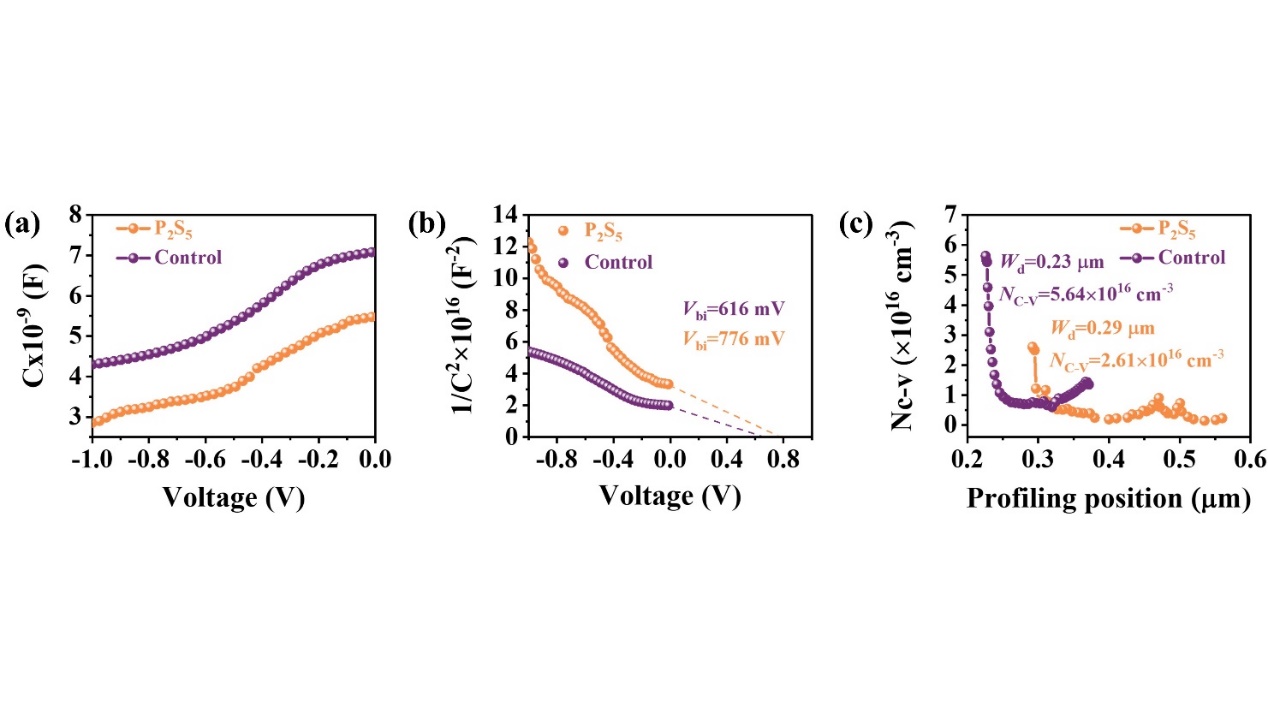


**Figure S13.** (a) *C*-*V* curves. (b) Built-in potential. (c) Plots of the *N*_CV_ vs. *W*_d_.


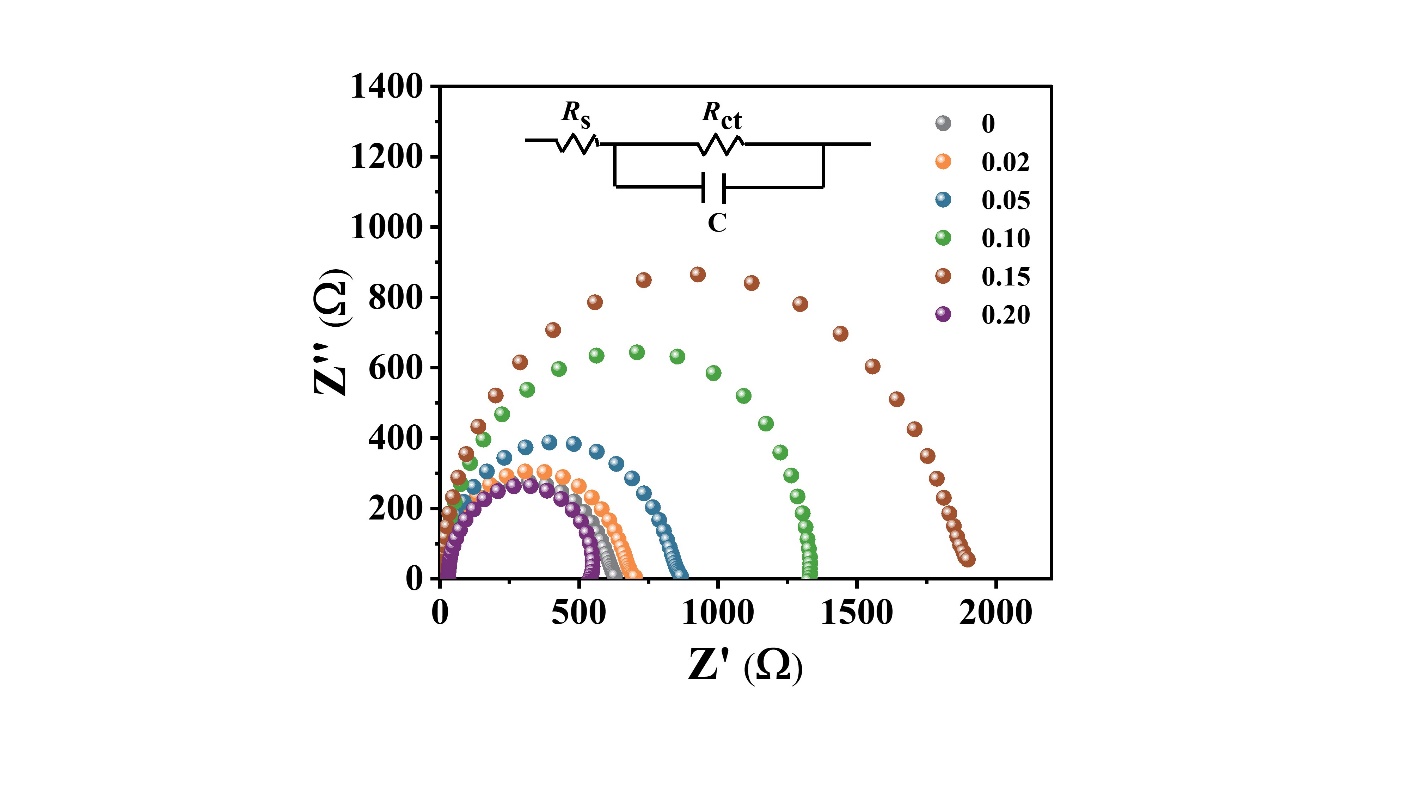


**Figure S14.** Nyquist plots of ACZTSSe devices under 0, 0.02, 0.05, 0.10, 0.15 and 0.20 mg/mL P_2_S_5_ concentrations.

**Table S1.** Photovoltaic parameters of the ACZTSSe solar cells with different concentrations of P_2_S_5_ from 0 to 0.20 mg/mL. The statistical data were obtained from 20 individual cells for each kind of device.

| P_2_S_5_ (mg/mL) |  | *J*_SC_ (mA/cm^2^) | *V*_OC_ (mV) | FF (%) | PCE (%) |
| --- | --- | --- | --- | --- | --- |
| 0 | Champion | 37.24 | 481.99 | 62.86 | 11.28 |
|  | Average | 37.15±0.52 | 482.69±2.39 | 62.19±0.81 | 11.15±0.12 |
| 0.02 | Champion | 37.96 | 504.86 | 63.79 | 12.23 |
|  | Average | 37.40±0.56 | 502.64±3.38 | 63.26±0.86 | 11.87±0.21 |
| 0.05 | Champion | 38.25 | 511.36 | 66.39 | 12.99 |
|  | Average | 37.42±0.63 | 511.89±3.69 | 65.88±1.04 | 12.54±0.22 |
| 0.10 | Champion | 38.26 | 516.83 | 66.76 | 13.20 |
|  | Average | 37.69±0.64 | 516.63±3.11 | 66.31±1.06 | 12.81±0.30 |
| 0.15 | Champion | 38.71 | 533.89 | 69.50 | 14.36 |
|  | Average | 38.44±0.41 | 525.97±5.07 | 68.64±1.00 | 13.74±0.40 |
| 0.20 | Champion | 37.83 | 526.45 | 67.18 | 13.38 |
|  | Average | 37.19±0.91 | 523.12±2.61 | 66.15±0.94 | 12.77±0.33 |

**Table S2.** Comparison of device parameters of high-efficiency CZTSSe solar cells reported in recent years.

| Device | *V*_OC_ (mV) | *J*_SC_ (mA/cm^2^) | FF (%) | PCE (%) | *E*_g_  (eV) | *V*_OC_/*V*_OC_ ^SQ^ (%) | *V*_OC_ deficit (V) | Ref. |
| --- | --- | --- | --- | --- | --- | --- | --- | --- |
| CZTSSe | 513.4 | 35.2 | 69.8 | 12.6 | 1.13 | 58.0 | 0.3736 | ^[8]^ |
| CZTSe | 490.8 | 37.37 | 68.19 | 12.5 | 1.037 | 61.5 | 0.3095 | ^[9]^ |
| CZTSSe | 499.0 | 36.4 | 68.0 | 12.4 | 1.04 | 62.2 | 0.3041 | ^[10]^ |
| CZTSSe | 529.0 | 33.7 | 72.9 | 13.0 | 1.11 | 61.0 | 0.3394 | ^[11]^ |
| CZTSSe | 547.2 | 34.3 | 70.0 | 13.14 | 1.11 | 63.1 | 0.3212 | ^[12]^ |
| CZTSSe | 551.2 | 35.7 | 71.7 | 14.13 | 1.097 | 64.5 | 0.3050 | ^[13]^ |
| CZTSSe | 520.0 | 39.08 | 63.32 | 12.86 | 1.077 | 62.3 | 0.3176 | ^[14]^ |
| CZTSSe | 546.0 | 35.86 | 65.85 | 12.89 | 1.14 | 61.0 | 0.3503 | ^[15]^ |
| CZTSSe | 540.0 | 34.6 | 72.3 | 13.5 | 1.074 | 64.8 | 0.2948 | ^[16]^ |
| CZTSSe | 546.0 | 35.9 | 69.4 | 13.6 | 1.10 | 63.7 | 0.3130 | ^[17]^ |
| CZTSSe | 513.85 | 38.69 | 69.27 | 13.77 | 1.047 | 63.7 | 0.2958 | ^[18]^ |
| CZTSSe | 541.8 | 39.11 | 66.14 | 14.01 | 1.094 | 63.6 | 0.3116 | ^[19]^ |
| CZTSSe | 576.0 | 36.9 | 70.0 | 14.9 | 1.10 | 67.1 | 0.2830 | ^[20]^ |
| This work | 533.89 | 38.71 | 69.50 | 14.36 | 1.032 | 67.2 | 0.2617 |  |

**Table S3.** Summary of TRPL fitting results for the control and P_2_S_5_ additive devices.

| Device | 𝜏_1_ (𝑛𝑠) | 𝐴_1_ (%) | 𝜏_2_ (𝑛𝑠) | 𝐴_2_ (%) | 𝜏_avg_ (𝑛𝑠) |
| --- | --- | --- | --- | --- | --- |
| Control | 1.79 | 35.21 | 7.76 | 64.79 | 7.09 |
| 0.15 mg/mL P_2_S_5_ | 2.23 | 30.20 | 8.95 | 69.80 | 8.30 |

**Table S4.** Summary of the fitting results derived from EIS measurement of ACZTSSe solar cells doped with 0, 0.02, 0.05, 0.10, 0.15, and 0.20 mg/mL P_2_S_5_ concentrations.

| Device | *R*_S_ (Ω) | *R*_ct_ (Ω) |
| --- | --- | --- |
| 0 | 19.50 | 641.04 |
| 0.02 | 14.61 | 613.22 |
| 0.05 | 12.93 | 787.66 |
| 0.10 | 10.14 | 1417.20 |
| 0.15 | 8.79 | 1856.00 |
| 0.20 | 30.57 | 531.92 |

**References**

[1] Z. Xu, Q. Gao, C. Cui, S. Yuan, D. Kou, Z. Zhou, W. Zhou, Y. Meng, Y. Qi, M. Ishaq, U. A. Shah, S. Wu, *Adv. Funct. Mater.* **2023**, *33*, 2209187.

[2] Y. Qi, N. Wei, Y. Li, D. Kou, W. Zhou, Z. Zhou, Y. Meng, S. Yuan, L. Han, S. Wu, *Adv. Funct. Mater.* **2024**, *34*, 2308333.

[3] G. Kresse, J. Furthmüller, *Comput. Mater. Sci.* **1996**, *6*, 15.

[4] G. F. Kresse, J., *Phys. Rev. B* **1996**, *54*, 11169.

[5] G. J. Kresse, D., *Phys. Rev.B* **1999**, *59*, 1758.

[6] J. P. B. Perdew, K.; Emzerhof, M. , *Phys. Rev. Lett.* **1996**, *77*, 3865.

[7] S. S. Hegedus, W. N. Shafarman, *Prog. Photovoltaics Res. Appl.* **2004**, *12*, 155.

[8] W. Wang, M. T. Winkler, O. Gunawan, T. Gokmen, T. K. Todorov, Y. Zhu, D. B. Mitzi, *Adv. Energy Mater.* **2014**, *4*, 1301465.

[9] J. Li, Y. Huang, J. Huang, G. Liang, Y. Zhang, G. Rey, F. Guo, Z. Su, H. Zhu, L. Cai, K. Sun, Y. Sun, F. Liu, S. Chen, X. Hao, Y. Mai, M. A. Green, *Adv. Mater.* **2020**, *32*, 2005268.

[10] Y. Gong, Y. Zhang, Q. Zhu, Y. Zhou, R. Qiu, C. Niu, W. Yan, W. Huang, H. Xin, *Energy Environ. Sci.* **2021**, *14*, 2369.

[11] Y. Gong, Q. Zhu, B. Li, S. Wang, B. Duan, L. Lou, C. Xiang, E. Jedlicka, R. Giridharagopal, Y. Zhou, Q. Dai, W. Yan, S. Chen, Q. Meng, H. Xin, *Nat. Energy* **2022**, *7*, 966.

[12] J. Wang, J. Zhou, X. Xu, F. Meng, C. Xiang, L. Lou, K. Yin, B. Duan, H. Wu, J. Shi, Y. Luo, D. Li, H. Xin, Q. Meng, *Adv. Mater.* **2022**, *34*, 2202858.

[13] J. Zhou, X. Xu, H. Wu, J. Wang, L. Lou, K. Yin, Y. Gong, J. Shi, Y. Luo, D. Li, H. Xin, Q. Meng, *Nat. Energy* **2023**, *8*, 526.

[14] M. Wang, H. Geng, J. Zhu, Y. Cui, S. Zhao, J. Fu, D. Kou, J. Sun, C. Zhao, S. Wu, L. Ding, Z. Zheng, *Adv. Funct. Mater.* **2023**, *33*, 2307389.

[15] Z. Yu, C. Li, S. Chen, Z. Zheng, P. Fan, Y. Li, M. Tan, C. Yan, X. Zhang, Z. Su, G. Liang, *Adv. Energy Mater.* **2023**, *13*, 2300521.

[16] Y. Gong, R. Qiu, C. Niu, J. Fu, E. Jedlicka, R. Giridharagopal, Q. Zhu, Y. Zhou, W. Yan, S. Yu, J. Jiang, S. Wu, D. S. Ginger, W. Huang, H. Xin, *Adv. Funct. Mater.* **2021**, *31*, 2101927.

[17] X. Xu, J. Zhou, K. Yin, J. Wang, L. Lou, M. Jiao, B. Zhang, D. Li, J. Shi, H. Wu, Y. Luo, Q. Meng, *Nat. Commun.* **2023**, *14*, 6650.

[18] L. Cao, L. Wang, Z. Zhou, T. Zhou, R. Li, H. Zhang, Z. Wang, S. Wu, A. Najar, Q. Tian, S. Liu, *Adv. Mater.* **2024**, *36*, 2311918.

[19] H. Wei, C. Cui, Y. Li, Z. Wu, Y. Wei, Y. Han, L. Han, B. Lu, X. Wang, S. Pang, Z. Shao, G. Cui, *Small* **2023**, *20*, 2308266.

[20] Y. Li, C. Cui, H. Wei, Z. Shao, Z. Wu, S. Zhang, X. Wang, S. Pang, G. Cui, *Adv. Mater.* **2024**, *36*, 2400138.
